# Identification of Time-Varying External Force Using Group Sparse Regularization and Redundant Dictionary

**DOI:** 10.3390/s23010151

**Published:** 2022-12-23

**Authors:** Huanlin Liu, Hongwei Ma

**Affiliations:** 1 School of Environment and Civil Engineering, Dongguan University of Technology, Dongguan 523808, China; 2 Guangdong Provincial Key Laboratory of Intelligent Disaster Prevention and Emergency Technologies for Urban Lifeline Engineering, Dongguan 523808, China; 3 School of Aerospace, Xi’an Jiaotong University, Xi’an 710049, China

**Keywords:** time-varying external force identification, inverse problems in vibration, group sparse regularization, redundant dictionary, standard *l*_1_-norm regularization, function expansion method

## Abstract

How to accurately identify unknown time-varying external force from measured structural responses is an important engineering problem, which is critical for assessing the safety condition of the structure. In the context of a few available accelerometers, this paper proposes a novel time-varying external force identification method using group sparse regularization based on the prior knowledge in the redundant dictionary. Firstly, the relationship between time-varying external force and acceleration responses is established, and a redundant dictionary is designed to create a sparse expression of external force. Then, the relevance of atoms in the redundant dictionary is revealed, and this prior knowledge is used to determine the group structures of atoms. As a result, a force identification governing equation is formulated, and the group sparse regularization is reasonably introduced to ensure the accuracy of the identified results. The contribution of this paper is that the group structures of atoms are reasonably determined based on prior knowledge, and the complexity in the process for identifying external force from measured acceleration responses is reduced. Finally, the effectiveness of the proposed method is demonstrated by numerical simulations and an experimental structure. The illustrated results show that, compared with the force identification method based on the standard *l*_1_-norm regularization, the proposed method can further improve the identified accuracy of unknown external force and greatly enhance the computational efficiency for the force identification problem.

## 1. Introduction

The accurate estimation of an unknown external force acting on a structure is a greatly important issue in several fields, such as structural reliability analysis and design [1,2], structural health monitoring [3,4,5,6], and fatigue life prediction [7,8]. However, it is difficult to directly measure the external force acting on the structure due to some practical issues, such as the high cost of instruments, difficulty in instrument installation, and so on [9,10,11]. Rather than directly measuring external force, structural responses can be more easily acquired. Therefore, many scholars pay attention to indirect force measurement techniques. As a useful technique, the force identification method can indirectly identify the external force from measured structural responses.

However, the force identification problem is a typical inverse problem, which means that the process of indirectly identifying unknown external force from structural responses is ill-posed. Thus, effective solution methods are important in this problem. In existing studies, the methods can be roughly divided into three categories [10,11,12,13,14,15,16], i.e., direct solution methods, regularization methods, and probabilistic/statistical methods, wherein regularization methods [11,12,13,14] often analyze the force identification problem based on deterministic methods and improve the ill-posedness of the original problem. Therefore, these methods are more popular in practical application, and different regularization methods have been introduced into the force identification problem.

In the early studies, several typical regularization methods are directly used to improve the ill-posedness, such as Tikhonov regularization, truncated singular value decomposition, generalized singular value decomposition, and so on [12,13,14,17]. For instance, as the most popular regularization method, Tikhonov regularization can preliminarily improve the identified results by penalizing the *l*_2_-norm of force vector with the Tikhonov matrix [10,13]. Many studies have been conducted on the application of the Tikhonov regularization in force identification problems [18]. However, the Tikhonov regularization method has some unavoidable limitations and disadvantages. For example, the approximate solution directly obtained from the Tikhonov regularization is too smooth, and the identified result may lack some details that the desired real solution might possess [19].

To further improve the identification accuracy, in the follow-up studies, some other regularization methods [18], such as the function expansion method [20,21,22], multiplicative regularization [23,24], sparse regularization [25,26,27,28], and so on [29,30,31,32], are introduced into the force identification problem. In particular, for the time-varying external force, it is usually not sparse in the time domain. Some regularization techniques, such as sparse regularization cannot be directly used in this problem. Meanwhile, for some other improved methods [29,30], the characteristics of time-varying force cannot be identified well. Thus, some scholars have paid attention to combining the function expansion method with a sparse regularization technique [25,26,27,33]. Based on the theory of function expansion method, the combination of discrete basis functions is represented as a dictionary, and each basis function is an atom in the dictionary. On the other hand, sparse regularization can sparsely select reasonable atoms from the dictionary to approximately express the characteristics of external force and simultaneously reduce the influences of noise [27]. For example, Qiao et al. [27] study the application of the standard *l*_1_-norm regularization with different dictionaries for both impact force identification and harmonic force identification problems, and the identified accuracy can be improved by comparing it with the traditional Tikhonov regularization method. However, the sparsity of solution obtained from the standard *l*_1_-norm regularization is considered at the independent atom level, the complexity in the process of this force identification method is high, and the solving process is time-consuming. Moreover, for time-varying external force identification, local identification results may be unsatisfactory.

Unlike the standard *l*_1_-norm regularization, the group sparse regularization method considers the sparsity of solutions in some group structures, which can reduce the complexity in the process of solving problems. In a special force identification problem, i.e., impact force identification, Qiao et al. [34] employs the prior information that the duration time of the actual impact force is quite short, and reasonable group sparsity of impact force in the time domain is considered, so group sparse regularization is reasonably introduced for impact force identification. Because the time-varying external force is usually not sparse in the time domain, the above prior information cannot be directly employed. How to introduce the group sparse regularization into the time-varying external force identification problem is an important work. If the group structures of atoms are not reasonable enough, the group sparse regularization method may not improve the identified accuracy of time-varying force. For example, the method in Ref. [35] directly uses the group number to determine the group structures of atoms; by comparing the identified results in Ref. [26], the identified accuracy of time-varying force component cannot be improved.

Considering the shortcomings above, in this study, a novel force identification method is proposed by reasonably introducing the group sparse regularization based on the prior knowledge in the dictionary. The way in which our work will contribute is by revealing the relevance of atoms in the given dictionary, and the group structures of atoms are reasonably determined for the group sparse regularization. As a result, the group sparse regularization can be reasonably introduced for identifying the time-varying external force. The complexity in the process of force identification is reduced, the computational efficiency is greatly enhanced, and the identified accuracy can be improved.

The structure of this paper is organized as follows. In Section 2, the basic theories are introduced and the proposed force identification method is explained in detail. Then, Section 3 provides several numerical validations on the proposed method, and the identified results are compared with those obtained from the standard *l*_1_-norm regularization. Furthermore, some experiments are carried out on a simply supported beam to validate the effectiveness of the proposed method in Section 4. Finally, some conclusions are drawn in Section 5.

## 2. Theoretical Background

### 2.1. Relationship between Structural Response and External Force

Firstly, an introduction about the force identification problem will be provided. For a linear time-invariant structure, it is assumed that the initial displacement and velocity responses are zeros, and *m* measurement points are used to acquire the structural responses. When a time-varying external force is acting on the structure, the relationship between the structural response at the *i*-th measurement point and the external force can be expressed as [10,12]:

(1) $$r_{i}\left(t\right)=\int_{0}^{t}h_{i}\left(t-\tau \right)f\left(\tau \right)d\tau \;$$

where $f\left(t\right)$ is the time-varying external force, $\begin{array}{cc} r_{i}\left(t\right) & \left(i=1,2,\ldots ,m\right) \end{array}$ is the structural response at the *i*-th measurement point, and $h_{i}\left(t\right)$ is the impulse response function and can be calculated based on the mode superposition method.

Because accelerometers are common sensors installed in the structure and the acceleration responses are usually used for force identification, the structural response $r_{i}\left(t\right)$ is considered as acceleration response in this study.

Assuming that *N* sampling points are acquired in a time interval [0, *T*] and the sampling time interval is Δt, with discretization, a set of algebraic equations between the acceleration response at the *i*-th measurement point and the external force can be obtained from Equation (1):

(2) $$\left\{\begin{array}{c} r_{i}\left(\Delta t\right)\\ r_{i}\left(2\Delta t\right)\\ \vdots \\ r_{i}\left(N\Delta t\right) \end{array}\right\}=\left[\begin{array}{cccc} h_{i}\left(\Delta t\right) & 0 & \cdots & 0\\ h_{i}\left(2\Delta t\right) & h_{i}\left(\Delta t\right) & \cdots & 0\\ \vdots & \vdots & \ddots & \vdots \\ h_{i}\left(N\Delta t\right) & h_{i}\left(N\Delta t-\Delta t\right) & \cdots & h_{i}\left(\Delta t\right) \end{array}\right]\left\{\begin{array}{c} f\left(\Delta t\right)\\ f\left(2\Delta t\right)\\ \vdots \\ f\left(N\Delta t\right) \end{array}\right\}$$

Equation (2) can be simply rewritten as:

(3) $$r_{i}=H_{i}f$$

where $r_{i}={\left\{\begin{array}{cccc} r_{i}\left(\Delta t\right) & r_{i}\left(2\Delta t\right) & \cdots & r_{i}\left(N\Delta t\right) \end{array}\right\}}^{T}$, $f={\left\{\begin{array}{cccc} f\left(\Delta t\right) & f\left(2\Delta t\right) & \cdots & f\left(N\Delta t\right) \end{array}\right\}}^{T}$, the transfer matrix $H_{i}\in {\mathbb{R}}^{N\times N}$ represents the relationship between acceleration response and external force vectors.

### 2.2. Function Expansion Method for Force Identification Problem

Because the time-varying external force is usually not sparse in the time domain, to effectively improve the ill-posedness of the force identification problem by using sparse regularization techniques, the function expansion method is adopted at first. As mentioned above, for the function expansion method, each basis function can be degraded as an atom, and the combination of these atoms is represented as a dictionary. Thus, the time-varying external force is represented by a dictionary $\Phi \in {\mathbb{R}}^{N\times L}$ and its coefficient vector $\alpha \in {\mathbb{R}}^{L\times 1}$, given as [20,21,22]:

(4) f≈Φα

where $\alpha ={\left\{\alpha_{1},\alpha_{2},\ldots ,\alpha_{L}\right\}}^{T}$, each column of the dictionary Φ is an atom, the *i*-th atom can be represented as $\varphi_{i}$, and the number of atoms is represented by *L*.

By substituting Equation (4) into Equation (3), the following equation is obtained:

(5) $$r_{i}=H_{i}\Phi \alpha =A_{i}\alpha$$

where $A_{i}=H_{i}\Phi$.

Because external force is difficult to be directly measured, it is necessary to indirectly identify external force from acceleration responses. If multiple accelerometers are used, a combination form of acceleration responses is considered as [11]:

(6) $$\left\{\begin{array}{c} r_{1}/{\left\|r_{1}\right\|}_{2}\\ \vdots \\ r_{m}/{\left\|r_{m}\right\|}_{2} \end{array}\right\}=\left[\begin{array}{c} A_{1}/{\left\|r_{1}\right\|}_{2}\\ \vdots \\ A_{m}/{\left\|r_{m}\right\|}_{2} \end{array}\right]\alpha$$

where ${\left\|•\right\|}_{2}$ is the standard Euclidean norm.

Similarly, Equation (6) can be simply rewritten as:

(7) r=Aα

where $r\in {\mathbb{R}}^{M\times 1}$, $A\in {\mathbb{R}}^{M\times L}$, and M=m×N.

The amplitude of time-varying force is usually different in each sampling point. Therefore, to arbitrarily express the time-varying force, the span of vector space based on the bases must be complete. That is to say, the bases in Equation (4) can be selected as some functions, such as wavelet function, cubic spline function, trigonometric function, rectangular function, and so on. Meanwhile, it should be ensured that the combination of these bases is a complete dictionary or redundant dictionary.

Compared with complete dictionaries, redundant dictionaries are more suitable for expressing the characteristics of time-varying force. Therefore, in this study, the dictionary Φ is designed as a redundant dictionary consisting of trigonometric and rectangular functions.

In detail, the expressions of sine and cosine functions in trigonometric functions are, respectively, given in Equations (8) and (9):

(8) $$\varphi_{sin}^{j}\left(t\right)=\begin{array}{cc} \sin\left[2\pi \left(j\Delta f\right)t\right] & j=1,2,\cdots ,N_{sin} \end{array}$$

(9) $$\varphi_{cos}^{j}\left(t\right)=\begin{array}{cc} \cos\left[2\pi \left(j\Delta f\right)t\right] & j=0,1,2,\cdots ,N_{cos} \end{array}$$

where Δf is the resolution of external force in the frequency domain, $N_{sin}$ and $N_{cos}$ are, respectively, represented by the numbers of atoms corresponding to sine and cosine functions, and they can be, respectively, calculated by Equations (10) and (11):

(10) $$N_{sin}=\left\lfloor \frac{f_{s}}{2.56\Delta f}\right\rfloor$$

(11) $$N_{cos}=\left\lfloor \frac{f_{s}}{2.56\Delta f}\right\rfloor +1$$

where $\left\lfloor •\right\rfloor$ is the floor function and $f_{s}$ is the sampling frequency.

The expression of rectangular functions is given in Equation (12):

(12) $$\begin{array}{cc} \varphi_{rect}^{j}\left(t\right)=\left\{\begin{array}{cc} 1 & \left(j-1\right)\times k_{rect}\times \Delta t\leq t<\min\left(j\times k_{rect}\times \Delta t,T\right)\\ 0 & others \end{array}\right. & j=1,2,\ldots ,N_{rect} \end{array}$$

where $k_{rect}$ is a positive integer, $\min\left(j\times k_{rect}\times \Delta t,T\right)$ represents the minimum value between $j\times k_{rect}\times \Delta t$ and T, and the number of atoms corresponding to rectangular functions is determined by Equation (13):

(13) $$N_{rect}=\left\lceil \frac{N}{k_{rect}}\right\rceil$$

where $\left\lceil •\right\rceil$ is the ceiling function.

When Δf and $k_{rect}$ are given, with discretization, the atoms of the redundant dictionary are calculated on the basis of Equations (8)–(13) with $\begin{array}{cc} {\left\|A\varphi_{i}\right\|}_{2}=1 & \left(i=1,2,\ldots ,L\right) \end{array}$ and $L=N_{sin}+N_{cos}+N_{rect}$.

Because the coefficients of some atoms may be influenced by noise, it is necessary to define the force identification problem with reasonable constraints, such as sparse regularization techniques.

### 2.3. Sparse Regularization Techniques for Force Identification Problem

Sparse regularization techniques have been developed in some excellent studies. When the dictionary expression of external force is considered, the function of the sparse regularization technique is to sparsely select reasonable atoms from the redundant dictionary and to approximately express the characteristics of external force. It can effectively reduce the influences of noise. In the following context, the force identification problems proposed based on standard *l*_1_-norm regularization and group sparse regularization techniques are, respectively, introduced.

#### 2.3.1. Force Identification Problem Based on Standard *l*_1_-Norm Regularization

Standard *l*_1_-norm regularization [28] is a popular technique for obtaining sparse solutions to inverse problems. By combining Equation (7) with standard *l*_1_-norm regularization, the *l*_1_-norm of the coefficient vector is adopted to define the force identification problem. As a result, an unconstrained optimization problem can be defined as:

(14) $$\alpha_{l_{1}}=\arg\underset{\alpha \in {\mathbb{R}}^{L\times 1}}{\min}\left\{\frac{\lambda }{2}{\left\|r-A\alpha \right\|}_{2}^{2}+{\left\|\alpha \right\|}_{1}\right\}$$

where ${\left\|•\right\|}_{1}$ represents the *l*_1_-norm of vector and λ is a weight coefficient. When λ→∞, the solution of Equation (14) converges to the solution obtained from the least squares method.

From Equation (14), it can be seen that the identified coefficient vector $\alpha_{l_{1}}$ is obtained by considering a balance between the residual norm ${\left\|r-A\alpha \right\|}_{2}^{2}$ and the *l*_1_-norm of vector α when the weight coefficient is given. Thus, the weight coefficient should first be determined for reasonable results, which will be introduced in the following section.

#### 2.3.2. Force Identification Problem Based on Group Sparse Regularization

Because the standard *l*_1_-norm regularization only considers the standard sparsity of the solution, the underlying structure sparsity of the solution cannot be revealed. The structure sparsity of the solution is directly related to the underlying relevance of atoms in the given dictionary. Therefore, the underlying relevance of atoms should be analyzed at first, and the underlying structure of atoms, such as group structure, joint structure, or tree structure, can be reasonably determined. As a result, corresponding structured sparse regularization techniques can be introduced to the specific problem. That is to say, how to determine the underlying structure of atoms is an important issue for the structured sparse regularization methods.

In this study, with the help of trigonometric and rectangular functions, the underlying relevance of atoms in the redundant dictionary is analyzed, and the group structures of atoms can reasonably be determined based on this prior knowledge.

More specifically, the sine and cosine functions given in Equations (8) and (9) can be used to express a Fourier series in an amplitude-phase form. Thus, when the frequency jΔf is given, the atom coefficients $\alpha_{2j}$ and $\alpha_{2j+1}$ have the following relationship:

(15) $$A_{j}\sin\left[2\pi \left(j\Delta f\right)t+\varphi_{j}\right]=\alpha_{2j}\sin\left[2\pi \left(j\Delta f\right)t\right]+\alpha_{2j+1}\cos\left[2\pi \left(j\Delta f\right)t\right]$$

where $A_{j}$ and $\varphi_{j}$, respectively, represent the *j*-th harmonic’s amplitude and its phase shift, the corresponding atom coefficients are $\alpha_{2j}=A_{j}\cos\varphi_{j}$ and $\alpha_{2j+1}=A_{j}\sin\varphi_{j}$, respectively.

Equation (15) indicates that the atoms corresponding to their coefficients $\alpha_{2j}$ and $\alpha_{2j+1}$ have obvious relevance. For each positive integer *j*, the atoms determined by Equations (8) and (9) are set as a group. When *j* = 0, the atom calculated by Equation (9) is set as an independent group.

On the other hand, the purpose of the rectangular functions defined by Equation (12) is used to approximate the local force components of the external force. In the time domain, each local force component can be considered as continuous in an extremely short time interval, so the atoms in each time interval are relevant, and they can be regarded as a group. Because the time interval for the local force component may be unknown in practice, the number of atoms $n_{rect}$ in each time interval is given directly in this study.

In summary, some atoms defined by Equations (8), (9) and (12) have obvious relevance. Therefore, by defining the group structures of these atoms, the structured sparse regularization technique is introduced for force identification with the redundant dictionary, and the group sparsity of solution can be achieved.

It is assumed that the atoms in the given redundant dictionary are divided into *p* groups based on the above prior knowledge, and the group sparse regularization [34,35,36] is introduced to define the minimum optimization problem:

(16) $$\alpha_{group}=\arg\underset{\alpha \in {\mathbb{R}}^{L\times 1}}{\min}\left\{\frac{\lambda }{2}{\left\|r-A\alpha \right\|}_{2}^{2}+\sum_{j=1}^{p}{\left\|\alpha_{g_{j}}\right\|}_{2}\right\}$$

where $g_{j}\left(j=1,2,\ldots ,p\right)$ is the *j*-th disjoint group and represents the atom numbers in the given dictionary. For the atoms in the same group, the corresponding coefficients tend to be zero or non-zero values simultaneously.

#### 2.3.3. Solution Algorithm for Force Identification Problems

It should be noted that when each atom in the given dictionary is degraded as a group, the standard *l*_1_-norm regularization can be rewritten as:

(17) $$\alpha_{l_{1}}=\arg\underset{\alpha \in {\mathbb{R}}^{L\times 1}}{\min}\left\{\frac{\lambda }{2}{\left\|r-A\alpha \right\|}_{2}^{2}+\sum_{j=1}^{L}{\left\|\alpha_{j}\right\|}_{2}\right\}$$

It can be observed that Equations (16) and (17) have the same expression forms. In this study, the split Bregman algorithm for structured sparsity (SBSS) is adopted to solve these two optimization problems, and the detail of this algorithm can be seen in Ref. [37]. The approximate solutions in Equations (16) and (17) can be, respectively, obtained after iterations, and both identification accuracy and computational efficiency in these two problems are compared in both numerical simulation and experimental validation.

**Figure 1:**
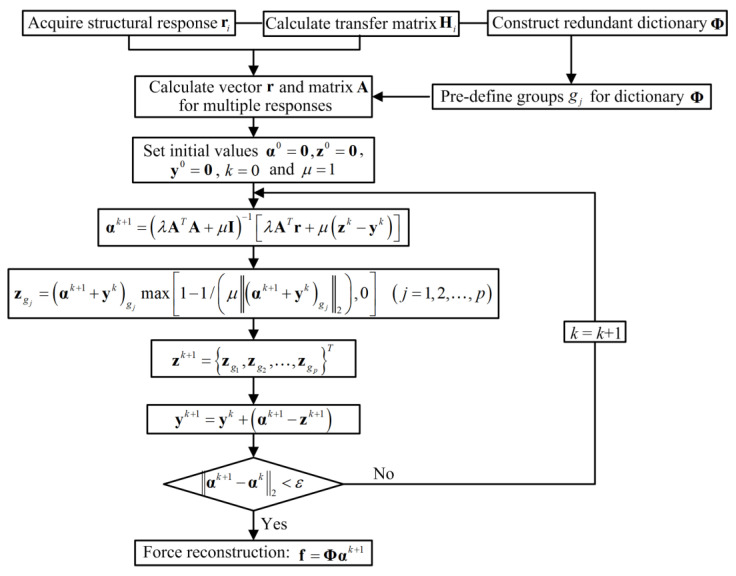
Basic flowchart of force identification method based on sparse regularization and redundant dictionary.

The basic flowchart for the force identification problem is shown in Figure 1. In Figure 1, for the group sparse regularization, ${\left(\cdot \right)}_{g_{j}}$ represents the elements of vector belonging to the *j*-th disjoint group $g_{j}$. For the standard *l*_1_-norm regularization, ${\left(\cdot \right)}_{g_{j}}$ is specified as ${\left(\cdot \right)}_{j}$, which is the *j*-th element of the vector, and p=L. ε is the tolerance error and is equal to 1 × 10^−6^.

## 3. Numerical Simulations

**Figure 2:**
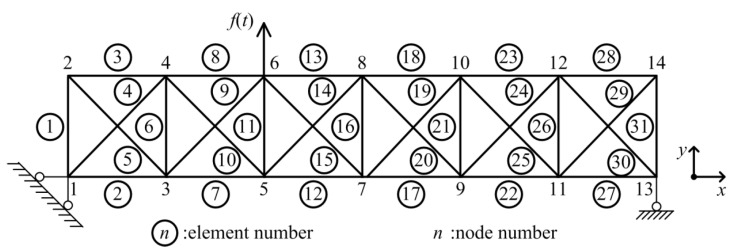
Geometric configuration of 31-bar planer truss.

To evaluate the effectiveness of the proposed force identification method, some numerical simulations are performed in a 31-bar planar truss [38], as shown in Figure 2. The sectional dimension is *b* × *h* = 0.05 m × 0.05 m with an elastic modulus of 70 GPa and a density of 2770 kg/m^3^. The length is 1.52 m for horizontal and vertical components. Rayleigh damping is adopted, and the first two damping ratios are assumed to be 1%.

A time-varying external force *f*(*t*) is acting in the *y* direction at node 6, the signal type of the external force is random excitation, which is considered as:

(18) f=100×randn      N

where randn is a vector, which is first drawn from the standard normal distribution, and then filtered by a low-pass Butterworth filter, which is designed with no more than 3 dB of ripple in a passband from 0 to 160 Hz, and at least 60 dB of attenuation in the stopband, defined as being from 319 Hz to the Nyquist frequency.

Based on Equation (2), the *i*-th elements, respectively, in the vectors f and randn have the following relationship:

(19) $$f\left(i\Delta t\right)=100\times randn_{i}$$

where $randn_{i}$ is the *i*-th element in the vector randn.

It is assumed that a few available accelerometers are used to acquire the acceleration responses, which are calculated based on the mode superposition method and the Newmark-β method. The first five frequencies of the planer truss, i.e., 36.4323 Hz, 76.0952 Hz, 133.8103 Hz, 223.3793 Hz, and 250.1825 Hz, are used in acceleration response calculation. The sampling frequency is *f_s_* = 640 Hz, and the sampling time is *T* = 1 s. As a result, the number of sampling points is *N* = 640.

For the redundant dictionary, the frequency resolution of the external force is Δf=1, and the positive integer $k_{rect}$ is set to 1. Therefore, based on Equations (10), (11) and (13), the number of atoms in the redundant dictionary can be calculated, i.e., $L=N_{sin}+N_{cos}+N_{rect}=251+250+640=1141$.

To simulate polluted acceleration responses, white noise is added to each calculated response:

(20) $$r_{i}^{n}=r_{i}+lev\times \sigma \left(r_{i}\right)\times noise$$

where $r_{i}$ and $r_{i}^{n}$ (i=1,2,…,m) are the *i*-th noiseless and noisy responses, respectively, lev is the noise level, $\sigma \left(\cdot \right)$ is the standard deviation of vector, and noise is a vector drawn from the standard normal distribution.

Furthermore, to estimate the identification accuracy, the relative percentage error (RPE) between true and identified external forces is defined as:

(21) $$RPE=\frac{{\left\|f_{true}-f_{iden}\right\|}_{2}}{{\left\|f_{true}\right\|}_{2}}\times 100\%$$

### 3.1. Comparative Study between Two Force Identification Methods

Firstly, the identified results, respectively, obtained based on Equations (16) and (17) are compared. The acceleration responses in the *y* direction of nodes 3 and 7 are used for force identification problems, and the noise level in Equation (20) is set to 5%. For the group sparse regularization, the group structures of the atoms are given as mentioned above, and the number of atoms $n_{rect}$ is set to 8. As a result, the number of groups is *p* = 331.

Because the SBSS is adopted for solving the optimization problems, in the iteration process of this algorithm, the weighted coefficient λ is needed. Meanwhile, as mentioned in the previous section, the weight coefficient λ is important for these two optimization problems. On the other hand, rather than selecting the weight coefficient, some posterior criteria are usually used to directly select the regularization parameter. Thus, based on Equation (16), an equivalent form is considered as:

(22) $$\alpha_{group}=\arg\underset{\alpha \in {\mathbb{R}}^{L\times 1}}{\min}\left\{\frac{1}{2}{\left\|r-A\alpha \right\|}_{2}^{2}+\beta \sum_{j=1}^{p}{\left\|\alpha_{g_{j}}\right\|}_{2}\right\}$$

where β=1/λ is the regularization parameter.

As a result, the regularization parameter β can be directly selected by some posterior criteria. In this study, the L-curve criterion is adopted to select the near-optimal regularization parameter. Because the time-varying external force is identified, the L-curve is considered as a plot in log–log scales of the residual norm of the solution ${\left\|r-A\alpha \right\|}_{2}$ versus the norm of the identified force ${\left\|f\right\|}_{2}$. By considering both computational efficiency and identification accuracy, the L-curve criterion is used twice to determine the parameter β. For an L-curve, the optimal regularization parameter $\beta_{opt}$ should lie on the corner of the L-curve. For values larger than the optimal regularization parameter, the residual norm of the solution ${\left\|r-A\alpha \right\|}_{2}$ increases without reducing the norm of the solution much. On the other hand, for values smaller than the optimal regularization parameter, the norm of the identified force ${\left\|f\right\|}_{2}$ increases rapidly without much residual decrease. Based on the above fact, in the first step, the range of regularization parameters that contains the optimal regularization parameter is determined. In the second step, the L-curve criterion is used to determine the near-optimal regularization parameter $\beta_{opt}$. As a result, based on the relationship between the weighted coefficient λ and regularization parameter β, i.e., β=1/λ, the weight coefficient is determined by the parameter $\beta_{opt}$. Similarly, the weight coefficient in Equation (17) is also selected in the same way. All calculations are run on a personal computer with an AMD Ryzen 7 2700 processor and 16 GB of memory.

**Figure 3:**
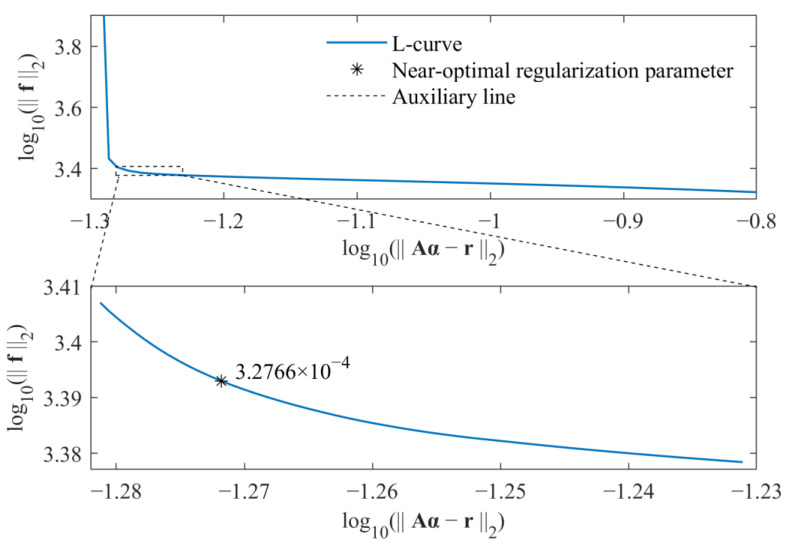
Selection of near-optimal regularization parameter for standard *l*_1_-norm regularization.

**Figure 4:**
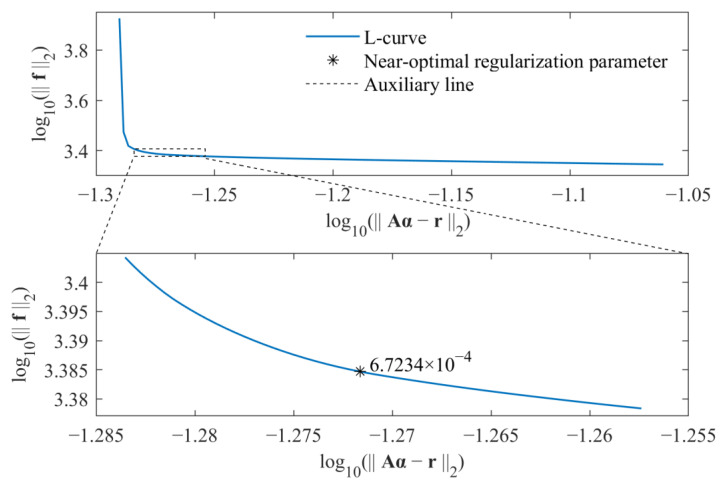
Selection of near-optimal regularization parameter for group sparse regularization.

The selections of near-optimal regularization parameters based on the standard *l*_1_-norm regularization and group sparse regularization are, respectively, shown in Figure 3 and Figure 4.

**Figure 5:**
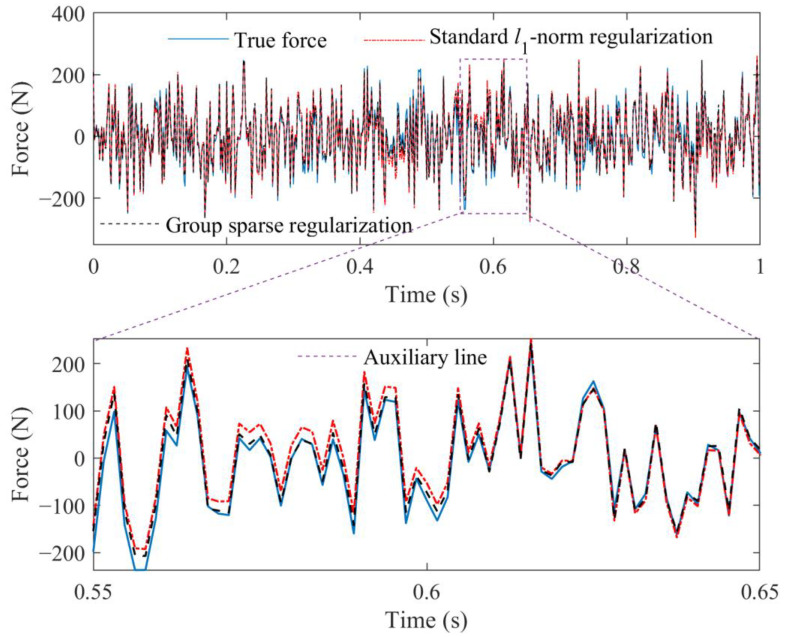
Identified result, respectively, obtained from standard *l*_1_-norm regularization and group sparse regularization.

For each figure, a range near to the L-curve corner is selected at first. Then, the maximum curvature of the L-curve in the selected range is determined based on the regularization tools developed by P. C. Hansen [39], and the corresponding parameter is selected as the near-optimal regularization parameter. Finally, the weight coefficient is calculated for corresponding force identification problem, and the external force can be identified from measured acceleration responses. The comparison of identified results obtained from these two methods is given in Figure 5.

From Figure 5, it can be seen that the external force identified by the proposed method is more accurate by comparing with that identified by the standard *l*_1_-norm regularization. The RPEs of the standard *l*_1_-norm regularization and group sparse regularization are 18.4109% and 15.7292%, respectively. Because the atom coefficients $\alpha_{2j}$ and $\alpha_{2j+1}$ have the relationship shown in Equation (15), the local identification accuracy of the time-varying external force can be further improved. These results indicate that the way to define group structures in this study is reasonable, which can improve.

On the other hand, with the help of group structures in the redundant dictionary, the group sparse regularization can obviously reduce the complexity in the process of the SBSS algorithm. In detail, when the near-optimal regularization parameters are, respectively, given to these two regularization techniques, the computation time for the standard *l*_1_-norm regularization and group sparse regularization techniques is 65.58 s and 7.63 s, respectively. That is to say, the group sparse regularization can also effectively enhance the calculated efficiency.

**Figure 6:**
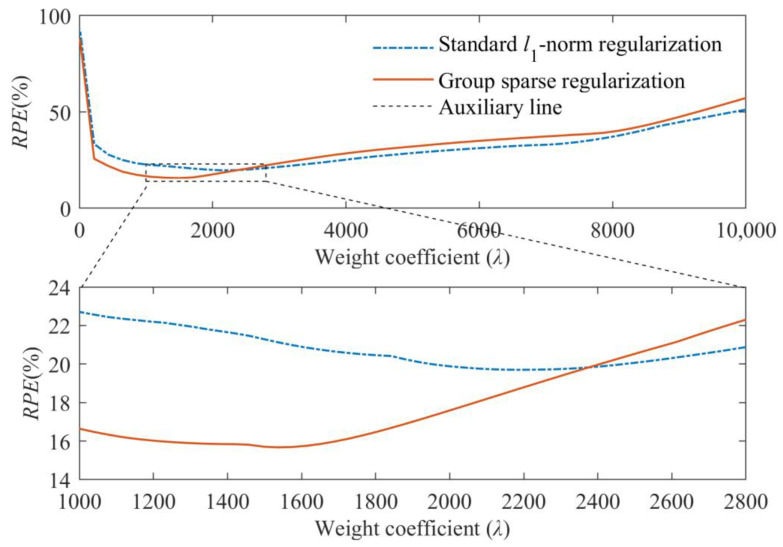
Comparative results between standard *l*_1_-norm regularization and group sparse regularization under different weight coefficients.

Moreover, the RPEs of identified results obtained from these two regularization techniques with different weight coefficients are shown in Figure 6. From Figure 6, it can be found that more accurate results can be achieved by the group sparse regularization when the optimal weight coefficient is used. Meanwhile, the minimum values of RPE curves are, respectively, approximate to the RPEs of identified results in Figure 5, which indicates that the selection of the weight coefficient with the help of L-curve criterion in this study is reasonable.

Therefore, when reasonable weight coefficients are, respectively, selected, the proposed force identification method based on the group sparse regularization can simultaneously enhance the identification accuracy and calculated efficiency by comparing with that based on the standard *l*_1_-norm regularization.

### 3.2. Influence of Different Noise Levels and Measurement Points

The noise levels and measurement points of acceleration responses are important parameters for the force identification problem, which will affect the results indirectly identified from structural responses. To further investigate these influences, three noise levels, i.e., *lev* = 1%, *lev* = 5%, and *lev* = 10%, for the structural responses at different measurement points are adopted. With the help of the weight coefficients selected by the L-curve criterion mentioned above, the RPEs are listed in Table 1.

From Table 1, it can be seen that when the measurement points are given, with the increase in noise levels, the weight coefficient is decreasing and the RPE is increasing. It indicates that the RPE of the identified result is positively correlated with the noise level of acceleration response. Meanwhile, it can be found that for the same noise level, the PREs of the identified results at different measurement points have small differences, which shows that the proposed method has great ability to solve the force identification problem under different application conditions.

In summary, the proposed method can further improve the force identification results compared with the standard *l*_1_-norm regularization. It is reasonable to consider the potential group structures of atoms in the given dictionary, so the proposed method can not only further improve the identification accuracy of the external force, but also significantly enhance the calculated efficiency. This way, defining objective function can effectively accord with the prior knowledge of atom relevance in the given dictionary.

## 4. Experimental Verifications

### 4.1. Experimental Setup

**Figure 7:**
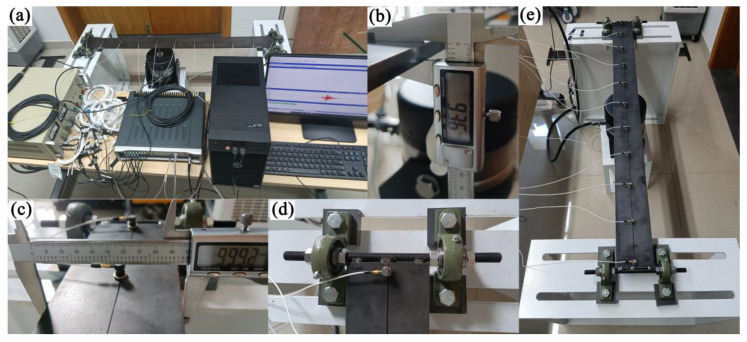
Experiment on beam structure for force identification. (**a**) Experimental setup. (**b**) Height of beam structure. (**c**) Width of beam structure. (**d**) Support of beam structure. (**e**) Beam structure.

**Figure 8:**
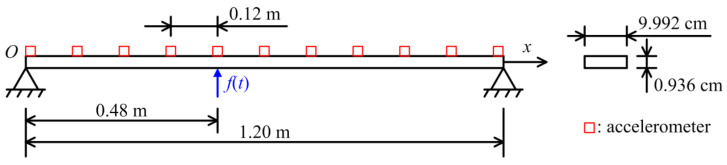
Experiment diagram of beam structure.

To evaluate the effectiveness of the proposed method, a beam with a span of 1.2 m was adopted for force identification in the laboratory, as shown in Figure 7. The two sides of the beam were simply supported, and the corresponding diagram is shown in Figure 8. The width and height of the beam were 9.992 cm and 0.936 cm, respectively. The elastic modulus was 2.0 × 10^11^ N/m^2^. Mode testing and signal acquisition were, respectively conducted in this study, so 11 accelerometers were evenly placed on the beam, and the distance of each two adjacent accelerometers was 0.12 m.

Firstly, mode testing of the beam structure was conducted. The external force is sine sweep signal, and it acted on the beam through the exciter. The sampling frequency was set as 160 Hz, and corresponding acceleration responses were acquired by the given accelerometers.

**Figure 9:**
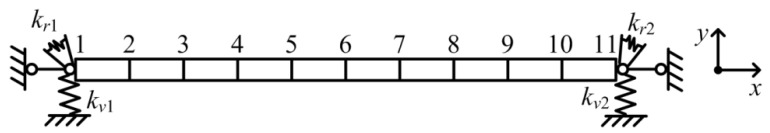
Finite element model of beam structure.

Modal parameters of the first three orders were analyzed, and these parameters were used to calibrate the finite element model (FEM) of the beam. Because the supporting conditions of the beam were not ideal, approximate supports in the FEM were used to simulate the support conditions, as shown in Figure 9. Meanwhile, because two threaded rods were, respectively used to connect the beam structure with the supports, the density of FEM needed to be updated. As a result, the vertical spring coefficients were set as *k_v_*_1_ = 1.0531 × 10^7^ N·m^−1^ and *k_v_*_2_ = 2.0495 × 10^7^ N·m^−1^, respectively. Meanwhile, the rotational spring coefficients were set as *k_r_*_1_ = 7.5103 × 10^1^ N·m/rad and *k_r_*_2_ = 6.2786 × 10^1^ N·m/rad, respectively.

**Figure 10:**
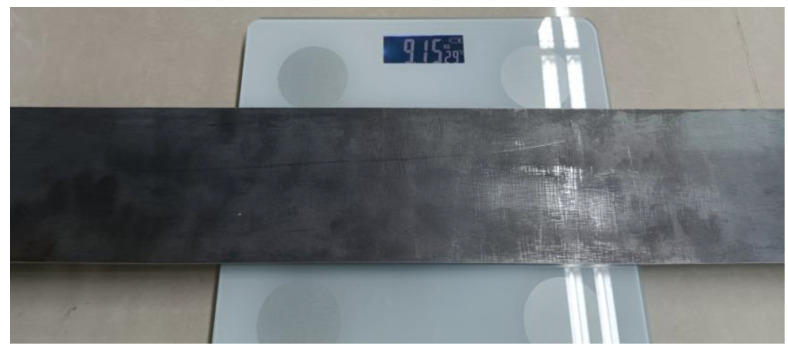
Mass of beam structure with threaded rods measured in laboratory.

The mass of the beam structure with threaded rods were measured in the laboratory, and it was equal to 9.15 kg, as shown in Figure 10. At the same time, according to the updating density of FEM and the geometric dimensions, the mass of the beam structure was equal to 8.98 kg, which was slightly smaller than the true value. This is because the length of connection between the beam structure and the threaded rods was smaller than the lengths of the threaded rods, and a part of the threaded rod was not taken as the part of beam structure. Therefore, the updating density of the FEM could be deemed as reasonable.

**Figure 11:**
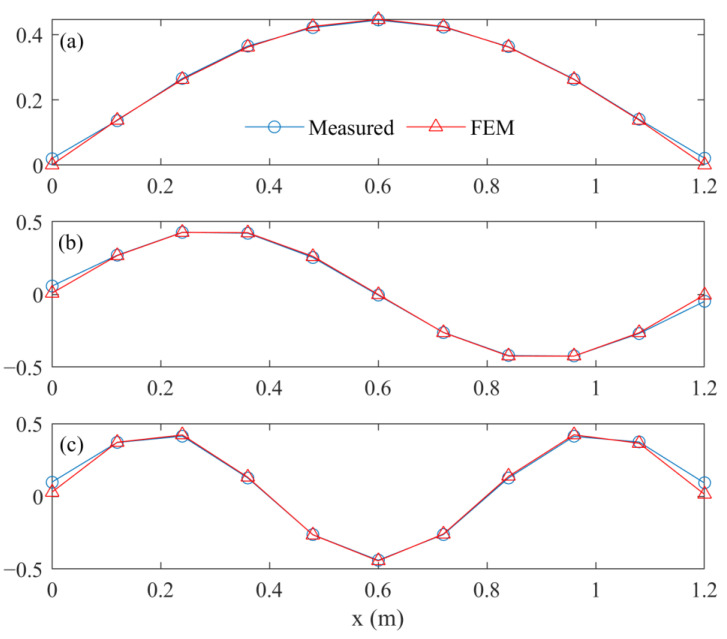
Comparison on first three mode shapes. (**a**) First mode. (**b**) Second mode. (**c**) Third mode.

As a result, the first three frequencies calculated based on the updating FEM were equal to the measured values when the approximate values were, respectively, considered as 14.8969 Hz, 58.8586 Hz, and 131.5050 Hz. At the same time, the first three damp ratios obtained from the mode testing results, i.e., 3.7136 × 10^−3^, 1.8644 × 10^−3^, and 3.8858 × 10^−3^, were directly used to calculate the transfer matrix. Moreover, the comparisons on the first three mode shapes are plotted in Figure 11. For intuitive comparison, each modal shape vector is scaled by its 2-norm.

The modal assurance criterion (MAC) values between measured mode shapes and the corresponding values obtained based on the FEM are 0.9991, 0.9955, and 0.9888, respectively. The updating mode parameters of the FEM are close to the measured values. It can be deemed that dynamic similarity between the FEM and the beam structure is achieved to a certain extent.

### 4.2. Verification of Proposed Method

First, white noise with a frequency range from 0 Hz to 80 Hz was selected as the excitation source, which acted in the *y* direction of node 5. It was assumed that the external force was difficult to be directly measured; measurement points different from the location of the external force were considered. Thus, the acceleration responses at the *y* direction of nodes 6 and 8 were acquired for force identification problem, and the measured time-varying external force was used for comparison with the identified results. For the group sparse regularization, the group structures of the atoms were given as mentioned above. Because the sampling frequency was 320 Hz, which was half of the sampling frequency in numerical simulations, the number of atoms $n_{rect}$ was set as 4.

**Figure 12:**
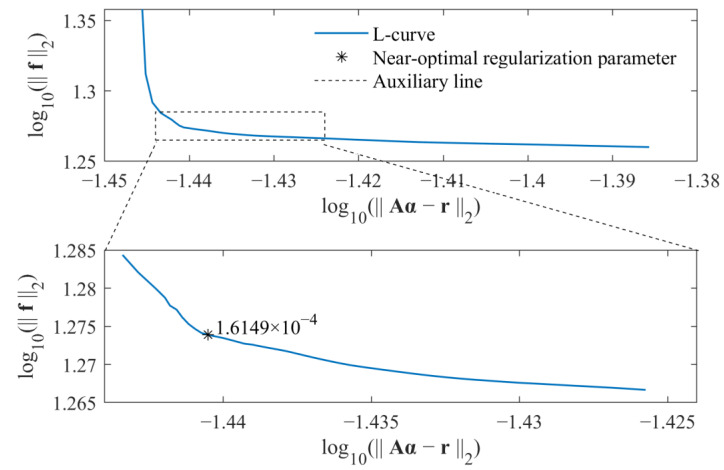
Selection of near-optimal regularization parameter for standard *l*_1_-norm regularization in experimental verifications.

**Figure 13:**
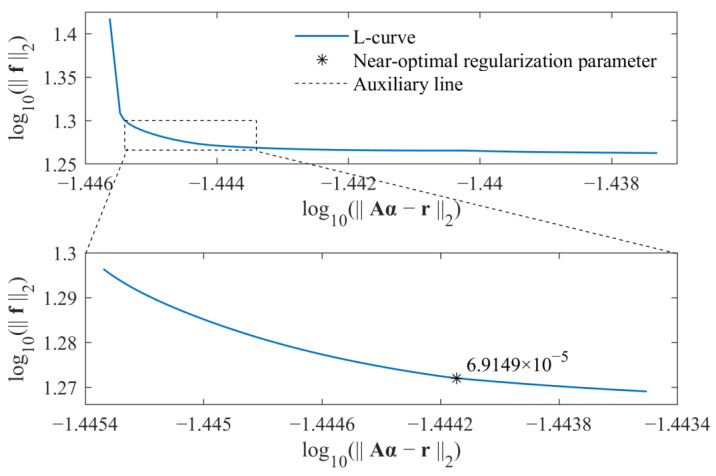
Selection of near-optimal regularization parameter for group sparse regularization in experimental verifications.

**Figure 14:**
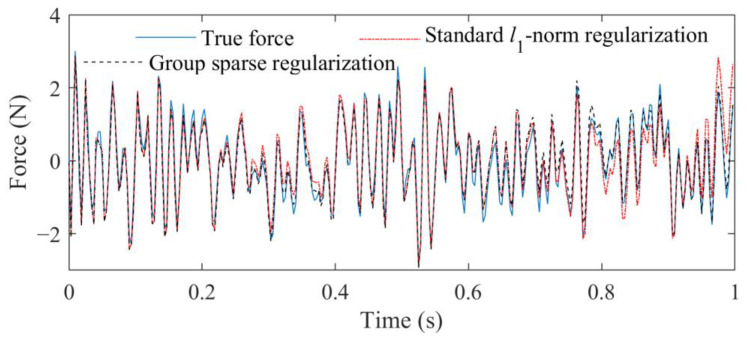
Identified result, respectively, obtained from standard *l*_1_-norm regularization and group sparse regularization in experimental verifications.

Similarly, the near-optimal regularization parameters for the standard *l*_1_-norm regularization and group sparse regularization were selected by the L-curve criterion, as, respectively, shown in Figure 12 and Figure 13. As a result, the weight coefficients were determined, and the identified external force obtained from the proposed method was compared with that obtained from the standard *l*_1_-norm regularization, as shown in Figure 14.

In Figure 14, the RPEs obtained based on the standard *l*_1_-norm regularization and group sparse regularization are 31.56% and 21.73%, respectively. Because the updated FEM was adopted for force identification, the errors in the transfer matrix were inevitable. In this situation, by adding the regularization constraint, defined based on the group structures, the local identification accuracy obtained by the proposed method is greatly improved. It indicates the group structures of atoms are reasonable enough when updated FEM is used. Meanwhile, the computation time for the standard *l*_1_-norm regularization and the proposed method is 16.35 s and 3.59 s, respectively.

Therefore, the same conclusions can be drawn as those given in Section 3.1. The proposed method can further improve the identification accuracy and enhance the calculated efficiency for the force identification problem.

**Figure 15:**
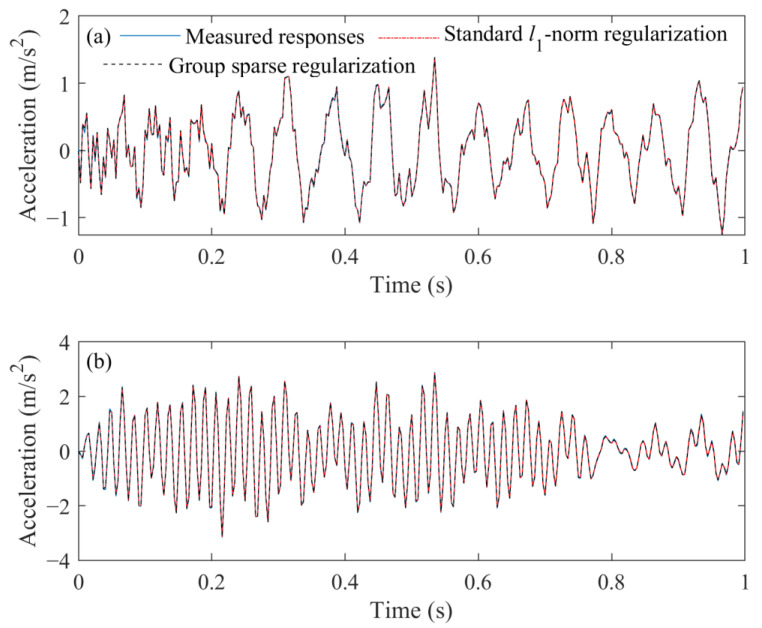
Comparison of measurement and reconstructed acceleration responses at different measurement points. (**a**) 1/2 span. (**b**) 7/10 span.

Furthermore, the acceleration responses reconstructed from the identified external force obtained from these two methods can be seen in Figure 15.

It can be observed that the measured acceleration responses at 1/2 span and 7/10 span are quite different. This is because the sampling frequency is 320 Hz, only the first three modes of beam structure are contained in the measured acceleration responses, and, as shown in Figure 11b, the node of the second mode is at the middle span of the beam structure. Meanwhile, it can be observed that the reconstructed acceleration responses obtained from different regularization methods are in good agreement with the measured responses in the time domain. It indicates that the identified results can be reasonably used to reconstruct structural responses.

To further study the application of the proposed method, several response combinations with different measured cases are adopted for force identification. The corresponding force identification results based on the proposed method are shown in Table 2.

In Table 2, it can be seen that despite the RPEs of the identified results fluctuating due to the influence of random noise and response combinations, the identified accuracy is acceptable. Therefore, when acceleration responses at a few measurement points are used, the proposed method can indirectly identify the time-varying external force with great identification accuracy.

## 5. Conclusions

In this paper, to indirectly measure time-varying external force from acceleration responses, a novel method based on the group sparse regularization is proposed. Firstly, with the help of trigonometric and rectangular functions, a redundant dictionary is constructed for the expression of external force. Then, the underlying relevance between the atoms is revealed, so the group structures of atoms in the redundant dictionary can be reasonably determined. Finally, the group sparse regularization is introduced based on the group structures, and the external force can be identified by solving the defined optimization problem. To evaluate the effectiveness of the proposed method, both numerical simulations and experimental verifications are carried out. Some conclusions are summarized as follows:

- The proposed method is an effective technique for indirectly measuring time-varying external force from acceleration responses in a few measurement points; both identified external forces and reconstructed responses are in good agreement with the measured values.
- Compared with the standard *l*_1_-norm regularization, the proposed method can further improve the force identification results in both numerical simulations and experimental verifications.
- The relevance of atoms in the redundant dictionary can obviously reduce the complexity in the process of force identification. When the same algorithm is used, the proposed method can enhance computational efficiency by comparing with the standard *l*_1_-norm regularization.

## Figures and Tables

**Table 1:** RPEs under influences of different noise levels and measurement points.

Measurement Points	Noise Level	RPE	Weight Coefficient (*λ*)
*y* direction of nodes 3 and 7	1%	10.3081%	1.6207 × 10^4^
*y* direction of nodes 2 and 8	1%	10.6728%	1.0780 × 10^4^
*y* direction of nodes 3 and 7	5%	15.7292%	1.4873 × 10^3^
*y* direction of nodes 3, 7 and 8	5%	17.8764%	9.5138 × 10^2^
*y* direction of nodes 2 and 8	5%	17.3825%	8.8676 × 10^2^
*y* direction of nodes 3 and 7	10%	24.1736%	8.7850 × 10^2^
*y* direction of nodes 2 and 8	10%	25.9328%	6.5278 × 10^2^

**Table 2:** RPEs under different measured cases and response combinations.

Cases	Response Combinations	RPE	Weight Coefficient (*λ*)
Case 1	1/2 & 7/10 span	21.7343%	1.4462 × 10^4^
Case 1	3/10 & 7/10 span	22.3612%	1.5461 × 10^4^
Case 1	3/10 & 1/2 span	17.5320%	1.0173 × 10^4^
Case 2	1/2 & 7/10 span	21.9778%	5.3531 × 10^3^
Case 2	3/10 & 1/2 span	19.8638%	1.0930 × 10^4^
Case 3	3/10 & 1/2 span	19.7285%	5.5294 × 10^3^

## Data Availability

Not applicable.
